# Gestational trophoblastic neoplasia mimicking ruptured ectopic pregnancy: A case report

**DOI:** 10.1097/MD.0000000000033947

**Published:** 2023-06-02

**Authors:** Su Zhen Jiang, Hong Xia Gong

**Affiliations:** a Department of Gynaecology, Dongguan Songshan Lake Tungwah Hospital, Dongguan, China.

**Keywords:** case report, ectopic pregnancy, gestational trophoblastic neoplasia

## Abstract

Gestational trophoblastic neoplasia (GTN) refers to the hydatidiform mole tissue that invades the myometrium or even penetrates the uterine wall to the broad ligament or abdominal cavity, and a few have distant metastases through blood transport. According to the World Health Organization^[1]^ 2020 (5th edition) classification lists an erosive hydatidiform mole as a borderline or biologically behavioral uncertain tumor, it continues to be clinically classified as a malignancy and combined with choriocarcinoma as a GTN. The clinical manifestations of GTN include amenorrhea, abnormal vaginal bleeding, and increased serum human chorionic gonadotropin level, which are also common clinical features of ectopic pregnancy. The diagnosis of typical GTN is not difficult. However, some patients with atypical clinical manifestations and a lack of specificity in their B-ultrasound images are easy to misdiagnose, especially when the lesions are located in special parts outside the uterus and lack specific imaging features.

**Patient concerns:**

A 41-year-old woman who presented 3 months after having an abortion with severe abdominal pain that lasted 15 hours.

**Diagnoses:**

CT showed massive blood accumulation in the abdominal cavity and the pelvic cavity. Uterine lesions? Transvaginal uterine ultrasound reveals: a right intrauterine mixed mass (approximately 83 * 66 mm mixed echo mass), a possible pregnancy, and a rupture pregnancy (right pregnancy). abdominal effusion (large) and clots, maximum front and rear diameters of 95 mm, pelvic effusion, and about 20 mm deep. HCG levels in the blood were 17,452 IU/L and hemoglobin levels were 81 g/L. Admission diagnosis: Abdominal pain investigation: ectopic pregnancy? Bleeding shock.

**Interventions:**

Laparoscopy and laparotomy followed by hysterectomy, treated by chemotherapy.

**Outcomes:**

Hysterectomy was required due to intraoperative hemostasis difficulties, and the patient lost her uterus forever.

**Lessons:**

Continued reporting of these cases are important so that the gynecologists are aware about the possibility of ruptured invasive mole and it should be kept as a differential diagnosis in all the pregnant women presents with acute onset lower abdominal pain.

## 1. Introduction

Gestational trophoblastic neoplasia (GTN) is a group of diseases caused by the abnormal proliferation of trophoblast cells in the placenta, which include benign mole and malignant trophoblast diseases; GTN can be secondary to any type of pregnancy, but has a low incidence ranging from 1 to 3/1000 pregnancies for hydatidiform mole to (1–9)/40,000 pregnancies for choriocarcinoma^[2]^; The incidence of ectopic pregnancy (EP) is 1% to 2%.^[3]^ In particular, GTN patients with extrauterine lesions are more likely to be misdiagnosed with EP. GTN is mainly treated with chemotherapy, while EP is treated with surgery or drug therapy, and misdiagnosis will lead to an incorrect treatment strategy for patients. Timely diagnosis and diagnosis of gestational trophoblastic tumors are the key factors to avoid unnecessary surgery. We present a case of invasive mole which gradually eroded in the uterus and the uterine vasculature, leading to sudden rupture of uterus, massive hemoperitoneum and shock, mimicking EP. Share this case in order to reduce the probability of an erosion hydatidiform mole being misdirected as an EP and improve the diagnosis rate of an erosion hydatidiform mole.

## 2. Case report

**Figure 1.**
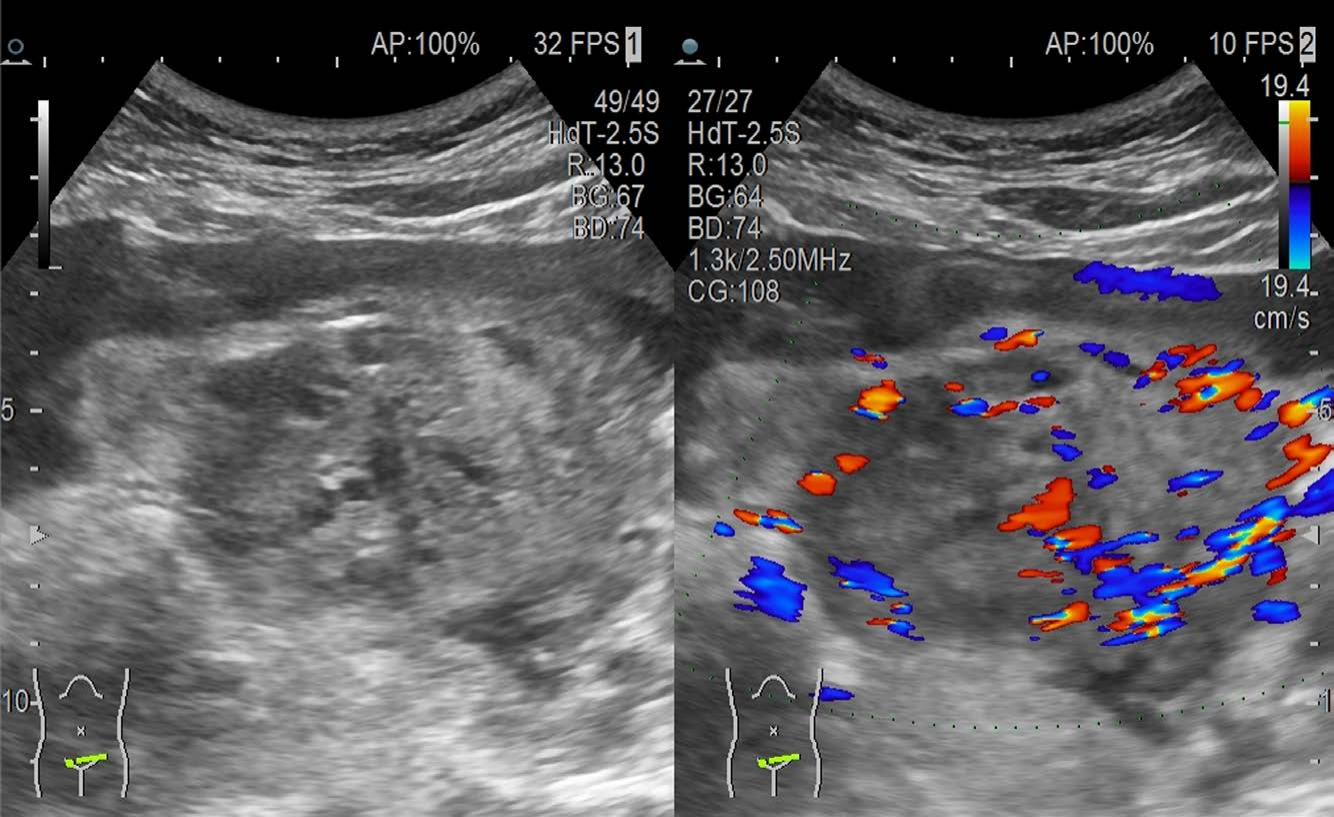
Ultrasound: Mixed mass on the right side of uterus, considering the possibility of ectopic pregnancy rupture, massive fluid accumulation in the pelvic cavity.

A 41-year-old woman presented to the emergency department 3 months after an abortion with sudden onset abdominal pain for 15 hours on April 16, 2020.The patient complained of having had an early induced abortion in the local hospital for an early pregnancy on January 14, 2020, and the intraoperative clearance object was not sent for pathological examination. Blood HCG was not reviewed after surgery due to recent severe travel difficulties with COVID-19. She had no sexual history for nearly 1 mouth. On April 25 PM, 2020, she complained of pain to syncope once, accompanied by nausea and vomiting 3 times, and the vomit was gastric content. The patient had regular menstruation, menarche at 13 years old, menstrual period 5 days, cycle 30 days, last menstrual November 28, 2019 menstrual volume normal, no dysmenorrhea, had been married, G4P3A1, had 2 previous vaginal births, 1 cesarean section in 2014, and 1 abortion. There were no special diseases in the past. There is no history of alcohol or drug abuse. No genetic history. Considerations for diagnosis in other hospitals “Abdominal pain checks cause: residual uterine horn pregnancy? “Intrauterine pregnancy tissue residue?” It was suggested that the patient be transferred to the superior hospital for further treatment. The patient was sent to our emergency department by ambulance from another hospital. Physical examination: temperature: 36.8°C, pulse 120 times per minute, breathing 20 times per minute, 80/50 mm Hg; conscious, passive position, painful face, poor examination, flat admission; abdominal tension, whole abdominal tenderness, positive rebound pain, mobile turbidity (+). The vulva development was normal, the vagina was unobstructed, there were no abnormal secretions inside, no odor, cervical lifting pain, or swing pain, according to a pelvic examination. The abdominal wall was thick, the patient had poor coordination, uterine tenderness, obvious tenderness in the bilateral appendage area, and no touch of the mass. CT showed massive blood accumulation in the abdominal cavity and the pelvic cavity. Uterine lesions? Transvaginal uterine ultrasound reveals: a right intrauterine mixed mass (approximately 83 * 66 mm mixed echo mass), a possible pregnancy, and a rupture pregnancy (right pregnancy). abdominal effusion (large) and clots, maximum front and rear diameters of 95 mm, pelvic effusion, and about 20 mm deep (Fig. 1). HCG levels in the blood were 17,452 IU/L and hemoglobin levels were 81 g/L. Admission diagnosis: Abdominal pain investigation: EP? Bleeding shock.

**Figure 2.**
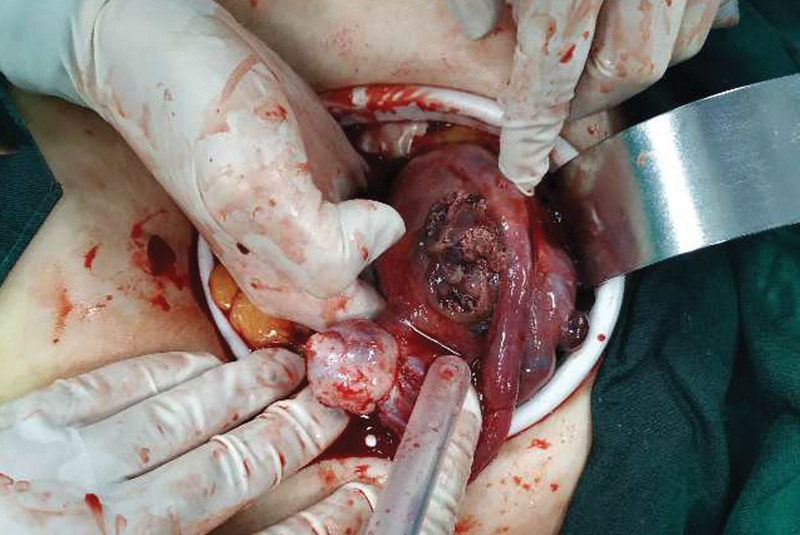
The right lateral wall of the uterus was obviously enlarged and uplifted near the uterine horn. The surface was filled with purplish blue earthworm shape. The scope was approximately 6*3cm. Rupture was visible on the surface, there were several blister-like tissues of varying sizes of the rupture, and there was active bleeding.

**Figure 3.**
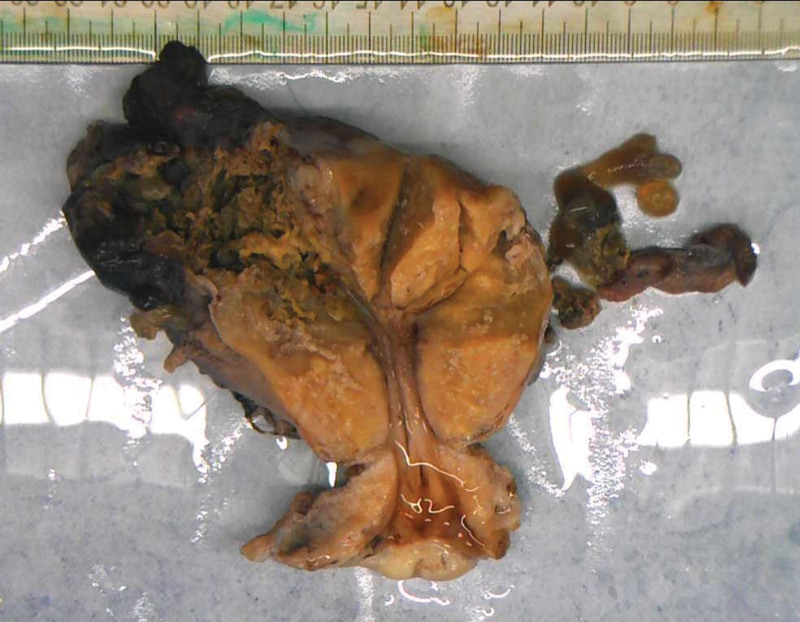
Uterus + right adnexa + left fallopian tube.

**Figure 4.**
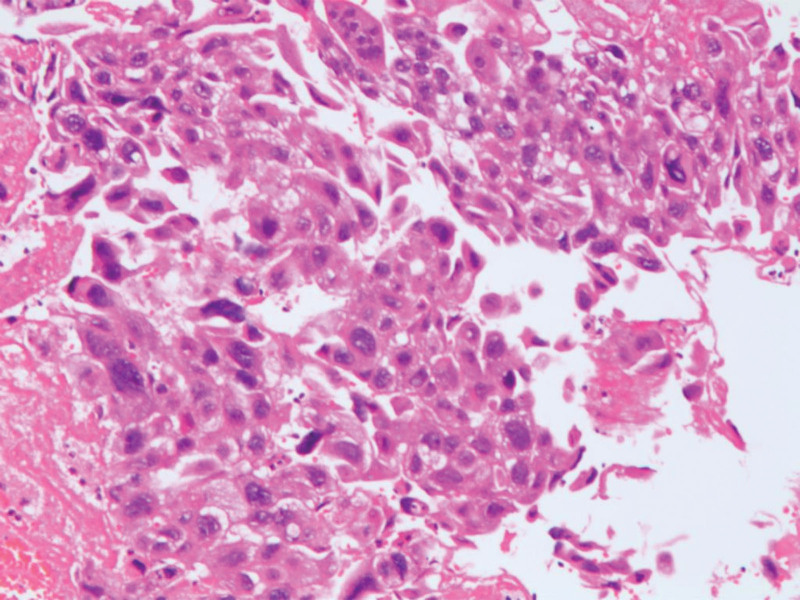
Excessive proliferation of trophoblast cells, visible infiltration of syncytial trophoblast cells and intermediate trophoblast cells into the uterine muscle layer, and invasion of the right adnexa, along with vascular invasion, and no invasion of the left fallopian tube (HE × 100).

Laparoscopic surgery was performed on the patient. Suction part blood and blood clots, exposed pelvic cavity, uterine enlargement, see the right uterus near the uterine horn bulge, the surface is purple blue earthworm filling, the right galloping tube lower mechanical and right broad ligament visible tablet hard nodules, surface is also purple blue, range about 6 * 3 cm, the right upper sacral ligame Laparotomy was immediately transferred, and laparoscopy exploration was moved to open total hysterectomy + right adnexectomy + left salpingectomy; When the uterus was pulled, a purple blue earthworm filled and ruptured the right uterine horn, and several blister-like tissues of various sizes overflowed.(Fig. 2). The procedure went smoothly, with approximately 600 mL of bleeding and an intraoperative blood gas analysis showing hemoglobin 58 g/L; In consideration of severe anemia, 7.5 IU of suspension and 600 mL of fresh frozen plasma were injected. Blood HCG of 7562.00 IU/L was reviewed on the first postoperative day, and blood HCG of 1154.00 IU/L was reviewed on the 6th postoperative day. Postoperative pathology (Figs. 3 and 4): (Right side attachment + uterus + left fallopian tube),excessive proliferation of trophoblast cells, visible infiltration of syncytial trophoblast cells and intermediate trophoblast cells into the uterine muscle layer, invasion of the right adnexa, along with vascular invasion, and no invasion of the left fallopian tube. Postoperative chest CT showed no metastasis. According to FIGO 2018, patient lesions were confined to the genitals and chest CT, and were classified as stage II^[2]^; Diagnosis: erosive hydatidiform mole (II: 7), be a high-risk patient, The patient was treated with standard chemotherapy and discharged from the hospital after being cured. There was no abnormality after 2 years of follow-up.

Approval for the study protocol was not necessary because our institutional review board does not require approval for case reports. We obtained written informed consent for treatment of the patient.

## 3. Discussion

A review of domestic and foreign literature found that GTN misdiagnosed as EP was reported in few literatures. Only 65 cases of gestational trophoblast cells being mistaken for EP have been reported in the medical literature; 11 cases were uterine perforation of pregnant trophoblast cells, and 8 cases were hysterectomy, permanently losing the possibility of the uterus and reproduction. Our case is in line with these 8 cases because delayed diagnosis leads to surgery and hysterectomy. but we succeeded in saving the live of the patient. With the development of hydatidiform moles, early diagnosis and early treatment are the keys to improving the cure rate. In this case, there is no chance of early treatment; COVID-19 is widely spread worldwide, seriously affecting the daily lives and health of humans. However, the impact of COVID-19 patients on other human diseases besides respiratory symptoms cannot be ignored^[4]^; Because the cases are so rare, it is unlikely that gynecologist sees such challenging cases in a lifetime; thus, reliance on ultrasound could lead to misdiagnosis; Therefore, when the patient has an acute clinical gynecological abdomen and severe bleeding, the patient should be asked about the medical history in detail^[5]^; HCG level normalized.

Within 14 weeks after evacuation in 95% of these patients.^[6]^There was no regular reexamination of blood HCG after surgery, which increases the difficulty of diagnosis in these patients. In addition to histology, molecular genetic studies can help in the diagnostic pathway. Earlier detection of molar pregnancy by ultrasound has resulted in changes in clinical presentation and decreased morbidity from uterine evacuation^[7]^; Pathological examination and color ultrasound review are critical. Surgery may have an important role in the management of GTN. Hysterectomy can be considered in uncontrolled uterine bleeding,^[7]^ So our case is in line with hysterectomy.

## 4. Conclucion

Gestational trophoblastic disease has a potential for myometrial and vascular invasion, leading to uterine perforation and massive hemorrhage. Therefore, to avoid adverse consequences it is necessary to Pathological examination after abortion and regular reexamination of blood HCG. Continued reporting of these cases are important so that the gynecologists are aware about the possibility of ruptured invasive mole and it should be kept as a differential diagnosis in all the pregnant women presents with acute onset lower abdominal pain. Although this is a rare case, it is a single case report that lacks the support of statistical data, more cases and studies are still needed.

## Author contributions

**Conceptualization:** Su Zhen Jiang, Hong Xia Gong.

**Data curation:** Su Zhen Jiang.

**Formal analysis:** Su Zhen Jiang.

**Supervision:** Hong Xia Gong.

**Writing – original draft:** Su Zhen Jiang.

**Writing – review & editing:** Su Zhen Jiang, Hong Xia Gong.
